# Diabetes Caused by *Elastase-Cre*-Mediated *Pdx1* Inactivation in Mice

**DOI:** 10.1038/srep21211

**Published:** 2016-02-18

**Authors:** Sota Kodama, Yasuhiro Nakano, Koji Hirata, Kenichiro Furuyama, Masashi Horiguchi, Takeshi Kuhara, Toshihiko Masui, Michiya Kawaguchi, Maureen Gannon, Christopher V. E. Wright, Shinji Uemoto, Yoshiya Kawaguchi

**Affiliations:** 1 Department of Surgery, Kyoto University Graduate School of Medicine, 53 Kawahara-cho, Shogoin, Sakyo-ku, Kyoto 606-8507, Japan; 2 Department of Clinical Application, Center for iPS Cell Research and Application, Kyoto University, 53 Kawahara-cho, Shogoin, Sakyo-ku, Kyoto 606-8507, Japan; 3 Vanderbilt Developmental Biology Program, Department of Cell and Developmental Biology, Vanderbilt University, Nashville, TN 37232, USA; 4 Department of Medicine, Vanderbilt University School of Medicine, Nashville, TN 37232, USA

## Abstract

Endocrine and exocrine pancreas tissues are both derived from the posterior foregut endoderm, however, the interdependence of these two cell types during their formation is not well understood. In this study, we generated mutant mice, in which the exocrine tissue is hypoplastic, in order to reveal a possible requirement for exocrine pancreas tissue in endocrine development and/or function. Since previous studies showed an indispensable role for *Pdx1* in pancreas organogenesis, we used *Elastase-Cre*-mediated recombination to inactivate *Pdx1* in the pancreatic exocrine lineage during embryonic stages. Along with exocrine defects, including impaired acinar cell maturation, the mutant mice exhibited substantial endocrine defects, including disturbed tip/trunk patterning of the developing ductal structure, a reduced number of Ngn3-expressing endocrine precursors, and ultimately fewer β cells. Notably, postnatal expansion of the endocrine cell content was extremely poor, and the mutant mice exhibited impaired glucose homeostasis. These findings suggest the existence of an unknown but essential factor(s) in the adjacent exocrine tissue that regulates proper formation of endocrine precursors and the expansion and function of endocrine tissues during embryonic and postnatal stages.

The mature pancreas is composed of two functional components: exocrine and endocrine tissue. Both tissue types originate during embryonic organogenesis from a common pool of multipotent pancreatic progenitors located within the pancreatic buds. Previous gene knockout studies have identified several crucial transcription factors in pancreas development. Neurogenin 3 (*Ngn3*)-null mice exhibit a lack of differentiated endocrine cells and an absence of endocrine transcription factors including Isl1, Pax4/6 and NeuroD^1^. Further, inactivation of *NeuroD* results in a significant reduction in endocrine cell numbers and impaired islet formation^2^,^3^. It has also been demonstrated that *Ptf1a*-null mice completely lack exocrine acinar cells but have a small number of endocrine cells^4^,^5^.

Although these studies have demonstrated that these genes function in the specification/differentiation of particular cell types during pancreatogenesis, there remains limited understanding about the degree of interplay between endocrine and exocrine development. We previously reported that reduced *Ptf1a* dosage resulted in remarkably reduced branching of the ductal tree with delayed specification/differentiation of acinar cells and pancreatic hypoplasia^6^. Interestingly, in hypomorphic *Ptf1a* mutants, the timing of endocrine cell differentiation was normal, but the total number of insulin-producing β cells was substantially reduced and the structure of islets disturbed, resulting in impaired glucose homeostasis. These findings supported the hypothesis that exocrine pancreatic tissue functions as a matrix necessary for proper endocrine pancreas formation^4^. However, since Ptf1a is expressed in the precursors of both acinar and endocrine cells^5^,^6^, we could not determine whether the endocrine defects observed in the hypomorphic *Ptf1a* mutants were cell-autonomous effects within the endocrine lineage or secondary effects of impaired exocrine formation.

Pancreatic and duodenal homeobox1 (*Pdx1*), which is the causative gene of maturity-onset diabetes of the young 4 (MODY4)^7^,^8^, regulates the transcription of genes involved in glucose homeostasis, such as insulin, glucokinase and glucose transporter type 2 (GLUT2), in adult β cells^9^,^10^,^11^. Developmentally, Pdx1 expression is first detected at approximately embryonic day 8.5 (E8.5) in the undifferentiated posterior foregut endoderm in mice^12^. As development proceeds, its expression expands to a wider region, including the dorsal and ventral pancreatic buds, developing antral stomach, duodenum and the lower bile duct. During mid-to-late embryonic stages, its expression is increased in β cells, but decreased in exocrine acinar/duct cells and epithelial cells of the common bile duct and rostral duodenum. Gene inactivation studies have demonstrated pivotal roles for *Pdx1* during embryogenesis, as global *Pdx1* knockout results in pancreatic agenesis, a lack of Brunner’s glands and malformation of the major duodenal papilla^13^,^14^,^15^.

Since *Pdx1* is indispensable for the formation of pancreatic exocrine and endocrine cells during development, we expected that exocrine-specific inactivation of *Pdx1* would be an ideal way to generate exocrine-lacking or hypoplastic mutants in which we could test if exocrine tissue is required for proper endocrine formation and function. For this purpose, we performed *Elastase-Cre*-mediated *Pdx1* inactivation and analyzed the pancreatic phenotype and function. We demonstrate that the mutant mice showed not only exocrine defects, but also fewer endocrine precursors and endocrine cells with less proliferation and delayed maturation, resulting in impaired glucose homeostasis. These findings support the notion that the exocrine pancreas is required for proper endocrine development and function, and that normal development of the pancreas occurs in an interactive, coordinated manner between the two tissues.

## Results

### *Elastase-Cre*-mediated *Pdx1* inactivation causes pancreatic hypoplasia and growth retardation

First, we evaluated the specificity and efficiency of the *Elastase-Cre*-mediated recombination. Lineage tracing of *Elastase-Cre*-expressing cells in control mice (*Pdx1*^+/+^;*Elastase-Cre*;*ROSA26r* and *Pdx1*^*loxP/*+^;*Elastase-Cre*;*ROSA26r* mice) showed that most progeny of *Elastase-Cre*-expressing cells differentiated into acinar cells at P1, but a subpopulation was detected in small ductal structures contiguous with the acini (Fig. S1). The proportion of lineage-labeled cells in Cytokeratin(+) duct cells tended to decrease during late embryonic stages in *Pdx1*^*loxP/*+^;*Elastase-Cre*;*ROSA26r* mice (approximately 10% at E16.5, 3% at P1 and 4% at P7), suggesting that lineage-labeled cells in the terminal ducts retained the ability to differentiate to acinar cells even at late embryonic to neonatal stages. Extremely few endocrine cells were labeled at P1 (Fig. S1): 0.44–1.30% and 0.57–0.80% in *Pdx1*^+/+^;*Elastase-Cre*;*ROSA26r* and *Pdx1*^*loxP/*+^;*Elastase-Cre*;*ROSA26r* mice, respectively (*n* = 3; at least 40 islets and 4000 endocrine cells were counted per mouse). These observations indicate satisfactory efficiency and specificity of the *Elastase-Cre*-based recombination in the exocrine lineage and negligible *Cre* expression in the endocrine lineage.

**Figure 1:**
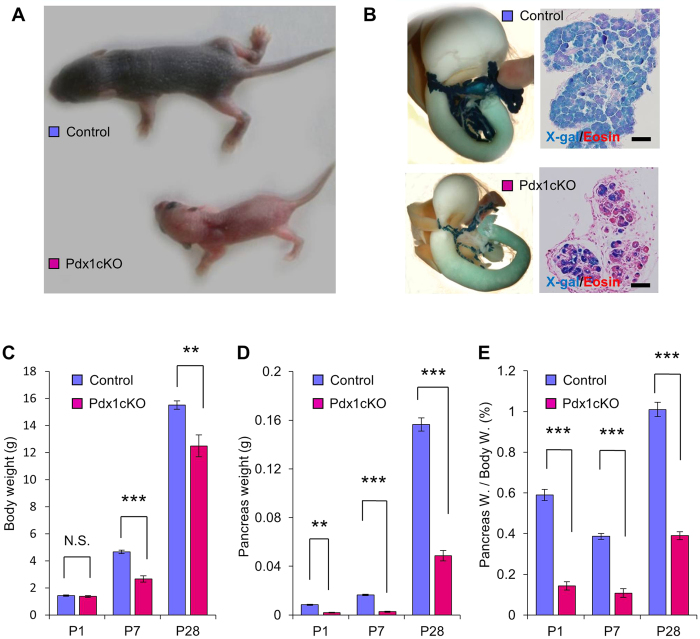
*Elastase-Cre*-mediated *Pdx1* inactivation causes growth retardation and pancreatic hypoplasia. (**A**) Gross appearance of mice at P7. Pdx1cKO mouse (bottom) is clearly smaller than its control littermate (top). (**B**) Macroscopic and histological view of the pancreas at P7. Note the X-gal stained, severely hypoplastic pancreas with poorly developed exocrine tissue in Pdx1cKO mouse (bottom). (**C**) Body Weight. Pdx1cKO mice (red) showed postnatal growth retardation. (**D**) Pancreas weight. Pancreas weight of mutant mice was approximately 21.4%, 16.6% and 31.0% that of control mice at P1, P7 and P28, respectively. (**E**) Percentage of pancreas weight/body weight. Scale bars, 50 μm. Bars represent the mean value ± SE. *P < 0.05, **P < 0.01, ***P < 0.001 (Pdx1cKO mice, *n* = 8 at P1, *n* = 4 at P7, *n* = 13 at P28; control mice, *n* = 7 at P1, *n* = 11 at P7, *n* = 5 at P28 in (**C–E**).

Newborn pups of Pdx1cKO mice (*Pdx1*^*loxP/*−^;*Elastase-Cre*;*ROSA26r* or *Pdx1*^*loxP/loxP*^;*Elastase-Cre*;*ROSA26r* mice) were indistinguishable from control littermates, but the body size of the mutant mice was clearly reduced by P7 (Fig. 1A). Mutants also had smaller pancreas and showed pancreatic hypoplasia at P7 based on histology (Fig. 1B). They exhibited growth retardation (Fig. 1C), and half did not survive to weaning. Pancreatic hypoplasia persisted in the survivors (Fig. 1D), and the pancreata of mutant mice were even smaller than expected from the reduced body size (Fig. 1E); the percentages of pancreas weight/body weight in the control mice were 0.61%, 0.39%, and 1.01% at P1, P7 and P28, respectively, while those in the mutants were 0.14%, 0.11% and 0.39% at P1, P7 and P28, respectively. Increased fat content in the stool of *Pdx1*^*loxP/−*^;*Elastase-Cre*;*ROSA26r* mice was detected, suggesting exocrine dysfunction and an associated decrease in lipid absorption by the small intestine. We could not detect X-gal(+) endocrine cells in Pdx1cKO mice at P1, strongly suggesting agenesis or the elimination of *Pdx1-*null endocrine cells during embryonic stages.

### *Elastase-Cre*-mediated *Pdx1* inactivation results in impaired exocrine differentiation during organogenesis

**Figure 2:**
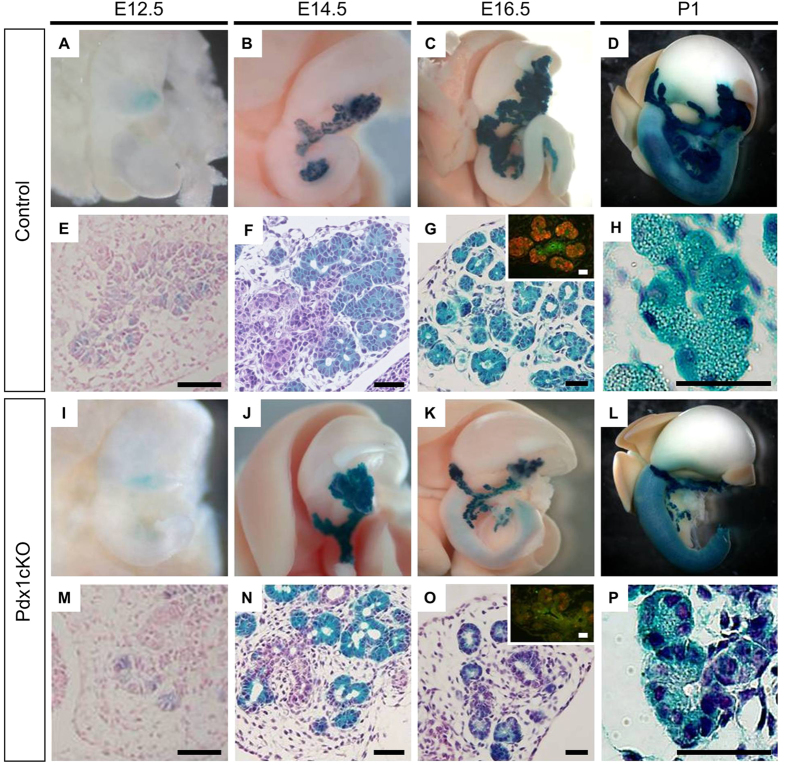
Impaired exocrine development in *Elastase-Cre*-mediated *Pdx1*-depleted mice. (**A–P**) Macroscopic and histological views. X-gal(+) progeny of *Elastase-Cre*-expressing cells were detectable as early as E12.5 (**A,E,I,M**). Pancreatic hypoplasia in Pdx1cKO mice was macroscopically apparent at E16.5 (compare **K** with **C**), but the histological phenotype was detected earlier. At E14.5, Pdx1cKO mice displayed reduced branching and dilated morphogenesis of the ductal tree (compare **N** with **F**). At E16.5, in control mice, the pancreatic ductal tree was well branched and many amylase-expressing cells were detected (**G**), but in Pdx1cKO mice branching of the ductal tree was reduced and amylase-expressing cells were fewer (**O**) (insets in (**G,O**) colabeled for amylase (red) and cytokeratin (green)). Hypoplastic pancreas persisted at P1 (compare **L** with **D**). In control mice, fully mature acinar cells were well developed, contained abundant exocrine granules with polarized subcellular localization of the nucleus on the basal side and had a low nucleus:cytoplasm ratio (**H**). In contrast, Pdx1cKO mice showed impaired acinar cytodifferentiation, and their acinar cells were smaller in size, had less polarized nuclear localization and a high nucleus:cytoplasm ratio (**P**). Scale bars, 50 μm.

Developmentally, X-gal(+) cells became detectable at E12.5 in the pancreatic epithelia of control and Pdx1cKO mice (Fig. 2A,E,I,M). Although some X-gal(+) cells in Pdx1cKO mice retained Pdx1 immunoreactivity at E13.5, Pdx1 expression was no longer detected in X-gal(+) cells at E14.5 or E16.5 (Fig. S2), showing that the Elastase promoter-driven Cre recombinase successfully activated the β-galactosidase gene at the ROSA26 locus and inactivated the *Pdx1* gene by E14.5. The expression of Pdx1 in some lineage-labeled cells at E13.5 may be explained by the relatively stable nature of the Pdx1 protein translated prior to gene recombination.

Until E14.5, there were no apparent macroscopic differences between the pancreata of control and Pdx1cKO mice (Fig. 2A,B,I,J), but the pancreata of the mutant mice was clearly smaller at E16.5 and did not recover afterwards (Fig. 2C,K). Histologically, exocrine development in the control mice resembled that of wild-type mice, with normal branching of the ductal tree at E14.5 and many amylase-expressing acinar cells detected at E16.5 (Fig. 2F,G). At P1, fully mature acinar cells were observed with basal nuclei, abundant cytoplasmic secretory granules and a low nucleus:cytoplasm ratio (Fig. 2H). In contrast, there was abnormal exocrine development in Pdx1cKO mice: despite no apparent macroscopic differences with control pancreata, the ductal tree was poorly branched and had a slightly dilated lumen at E14.5 (Fig. 2N). In addition, only few amylase-expressing cells were detected at E16.5 (Fig. 2O-inset) and acinar cytodifferentiation was extremely impaired, as acinar cells were much smaller and cuboidal, had few cytoplasmic granules, and a centrally located nucleus (Fig. 2P).

### *Elastase-Cre*-mediated *Pdx1* mutant mice exhibit impaired glucose homeostasis with reduced insulin secretion

**Figure 3:**
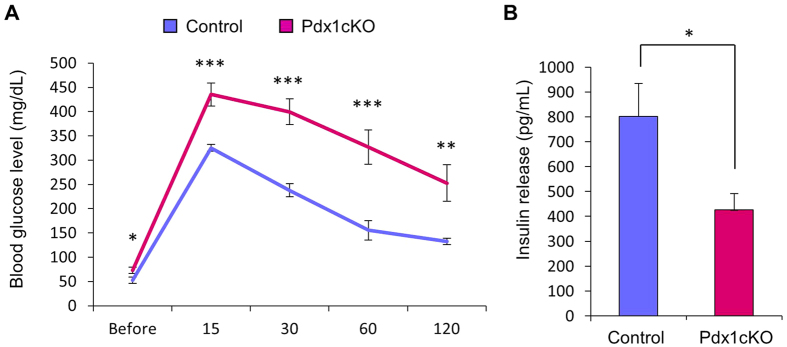
Impaired glucose homeostasis in Pdx1cKO mice. (**A,B**) Results of the intraperitoneal glucose tolerance test (IPGTT) and plasma insulin concentrations in Pdx1cKO (red) and control (blue) mice. IPGTT showed that Pdx1cKO mice represent impaired glucose homeostasis (**A**, Pdx1cKO mice, *n* = 9; control mice, *n* = 4) and have significantly lower insulin secretion 15 minutes after glucose challenge at P28 (**B**, Pdx1cKO mice, *n* = 4; control mice, *n* = 4). Bars represent the mean value ± SE. *P < 0.05, **P < 0.01, ***P < 0.001.

To test the hypothesis that disturbed exocrine development causes abnormal endocrine development and/or function, we compared Pdx1cKO mice (*Pdx1*^*loxP/loxP*^;*Elastase-Cre* mice) to *Pdx1*^+/+^;*Elastase-Cre* mice controls, because a dosage effect of *Pdx1* in endocrine function was previously reported^16^,^17^. Interestingly, the mutant mice were mildly hyperglycemic, and IPGTT demonstrated a significant impairment in glucose homeostasis in Pdx1cKO mice at P28 (Fig. 3A). Plasma insulin 15 min after glucose injection was significantly lower in mutants (Fig. 3B).

### Reduced proliferation and accelerated apoptosis in the developing ductal tree in *Elastase-Cre*-mediated *Pdx1* mutant mice

**Figure 4:**
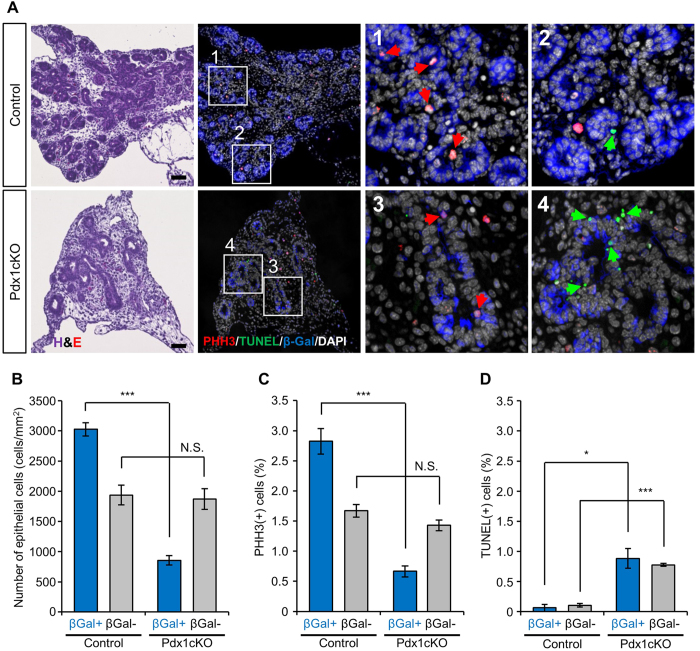
Reduced proliferation and accelerated autonomous and non-autonomous cell death in Pdx1cKO mice. (**A**) PHH3 and TUNEL analyses with β-gal staining of E14.5 pancreata. High magnification pictures of the indicated square are shown in the right two panels. Red and Green arrows represent PHH3(+) and TUNEL(+) cells, respectively. (**B-D**): Quantification of epithelial cells (**B**), PHH3(+) cells (**C**) and TUNEL(+) cells (**D**) at E14.5 (Pdx1cKO mice, *n* = 3; control mice, *n* = 4). In control and Pdx1cKO pancreata, β-gal(+) cells were detected in the tip of the branching ductal tree. Quantification analyses revealed that the number of lineage labeled cells was reduced in Pdx1cKO mice, while that of non-labeled cells remained the same as control (**B**). Pdx1-depleted, β-gal(+) cells proliferated less frequently and showed more apoptosis in Pdx1cKO mice (**C,D**). Note the significant TUNEL-positivity in the β-gal(−) population in Pdx1cKO mice, suggesting non-cell autonomous death. Scale bars, 50 μm. Bars represent the mean value ± SE. *P < 0.05, ***P < 0.001.

To gain insights on the mechanism responsible for impaired glucose homeostasis in Pdx1cKO mice, we analyzed embryonic pancreas formation. At E14.5, β-gal(+) cells were detected in the tip of the branching ductal tree in both control and Pdx1cKO mice (Fig. 4A). The number of lineage-labeled cells was significantly reduced in Pdx1cKO mice, while that of non-labeled cells remained the same as that in control mice at this stage (Fig. 4B). Consistent with the macroscopically smaller pancreas at E16.5, PHH3 and TUNEL staining revealed that the progeny of Pdx1-inactivated cells (β-gal(+) cells) in mutant mice proliferated less (Fig. 4C) and had increased apoptosis at E14.5 (Fig. 4D). Interestingly, while the percentage of PHH3(+) cells in the non-lineage labeled cells remained the same between control and mutant mice, we observed increased cell death of the β-gal(−) cells in mutant mice, suggesting non-cell autonomous effects within the developing ductal tree (Fig. 4C,D).

### Impaired tip/trunk patterning in *Elastase-Cre*-mediated *Pdx1* mutant mice

**Figure 5:**
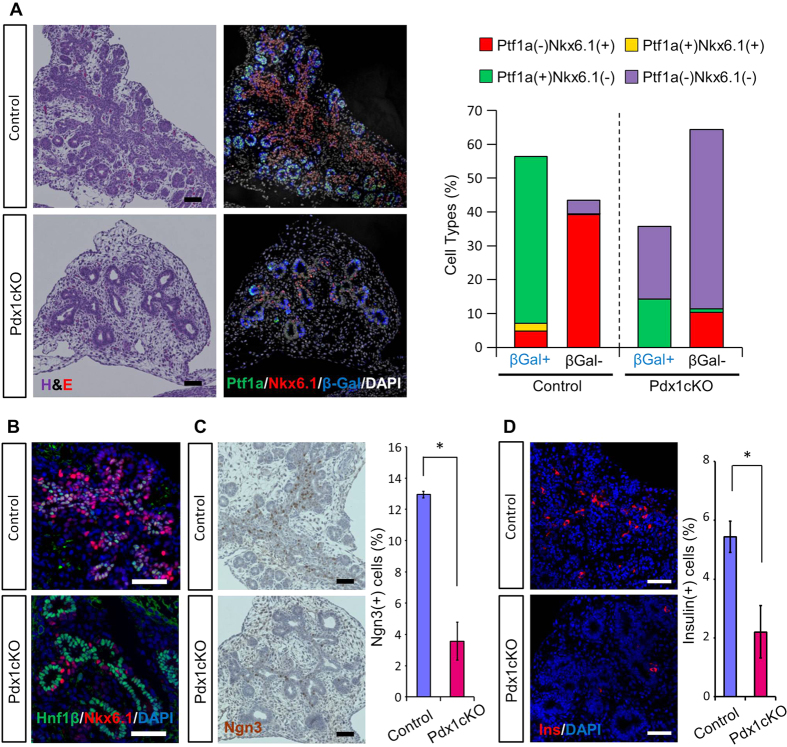
Impaired tip/trunk patterning caused reduced endocrine cells in embryonic Pdx1cKO pancreas. (**A**) Immunostaining of Ptf1a and Nkx6.1 pancreata and cell counting at E14.5. In control mice, the majority of β-gal(+) and β-gal(−) cells were composed of Ptf1a(+)Nkx6.1(−) and Ptf1a(−)Nkx6.1(+) populations, respectively, suggesting that *Elastase-Cre*-mediated markings are predominantly detected in the tip region. On the contrary, in Pdx1cKO mice, the percentage of Ptf1a(+)Nkx6.1(−) cells in the tip region and Ptf1a(−)Nkx6.1(+) cells in the trunk region were decreased while the percentage of the Ptf1a(−)Nkx6.1(−) population increased (Pdx1cKO mice, *n* = 4; control mice, *n* = 4). (**B**) Double staining of Nkx6.1 and Hnf1β at E14.5. Note that many cells coexpressed Nkx6.1 and Hnf1β in control mice, whereas the majority of epithelial cell expressed either Nkx6.1 or Hnf1β in mutants. (**C,D**) Immunostaining of Ngn3 (**C**) (Pdx1cKO mice, *n* = 3; control mice, *n* = 4) and insulin (**D**) (Pdx1cKO mice, *n* = 3; control mice, *n* = 3) and cell counting at E14.5. The percentages of Ngn3-expressing cells and insulin-producing cells were reduced in Pdx1cKO pancreata. Scale bars, 50 μm. Bars represent the mean value ± SE. *P < 0.05.

It was previously reported that the developing ductal tree gradually obtains tip/trunk patterning through which regional control of endocrine and exocrine differentiation is determined; endocrine cells originate from the Nkx6.1-expressing trunk domain, whereas exocrine acinar cells are differentiated from Ptf1a-expressing tip domain after E14^18^,^19^,^20^. Considering possible non-cell autonomous effects on cell survival within the developing ductal tree (Fig. 4D), we speculated that conditional inactivation of *Pdx1* by the *Elastase-Cre* transgene disturbs the tip/trunk patterning of the ductal tree to reduce the formation of endocrine precursors in Pdx1cKO mice. As shown in Fig. 5A, the majority of lineage-labeled and non-labeled cells in control mice at E14.5 were Ptf1a(+)Nkx6.1(−) tip cells and Ptf1a(−)Nkx6.1(+) trunk cells, respectively, suggesting that *Elastase-Cre-*mediated recombination occurred predominantly in the tip region. However, in mutant mice, not only was the percentage of Ptf1a-expressing cells reduced in the lineage-labeled tip region, but so too was the percentage of Nkx6.1(+) trunk cells. Reflecting this phenotype, the percentage of Ptf1a(−)Nkx6.1(−) cells was increased in the mutants. In addition, we found abnormal cell differentiation in the trunk region. At E14.5, ductal epithelium in the trunk region is composed of Hnf1β-expressing cells^21^. However, while most Hnf1β-expressing cells coexpressed Nkx6.1 in control mice, they did not in Pdx1cKO mutant mice (Fig. 5B). Thus, Ptf1a(−)Nkx6.1(−) cells, which were increased in the mutants, are suspected to be mainly composed of Hnf1β(+) cells. Finally, epithelial cells had a reduced percentage of Ngn3-expression and fewer β cells in mutant mice at E14.5 (Fig. 5C,D).

### Reduced proliferation and delayed maturation of endocrine cells is accompanied by islet disorganization in *Elastase-Cre*-mediated *Pdx1* mutant mice

**Figure 6:**
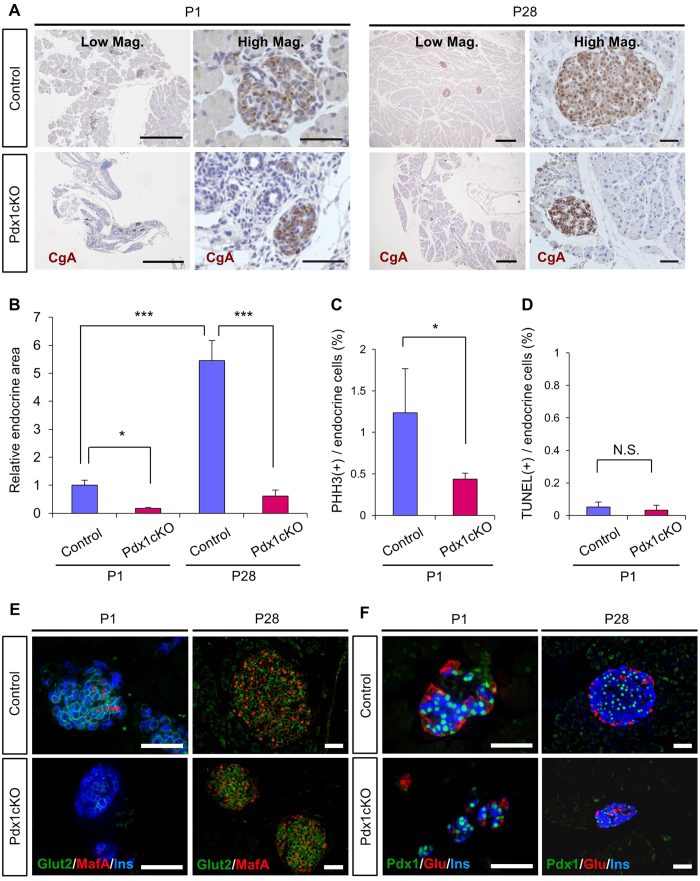
Reduced proliferation and delayed maturation of endocrine cells and impaired islet structure in postnatal Pdx1cKO mice. (**A,B**) Chromogranin-A (CgA) staining and relative endocrine area. Mutant mice had reduced CgA(+) endocrine cell content at P1 and P28. Note the extremely reduced postnatal endocrine expansion in Pdx1cKO mice (Pdx1cKO mice, *n* = 7 at P1, *n* = 6 at P28; control mice, *n* = 5 at P1, *n* = 4 at P28). (**C,D**) PHH3 staining and TUNEL staining at P1. The frequency of PHH3 positivity among endocrine cells was significantly lower in mutants (**C**) (Pdx1cKO mice, *n* = 5; control mice, *n* = 7), but TUNEL analysis showed no significant difference in apoptosis (**D**) at P1 (Pdx1cKO mice, *n* = 3; control mice, *n* = 4). (**E,F**): Histological sections immunolabeled for Glut2, MafA, Pdx1, glucagon (Glu) and insulin (Ins). Islets were smaller in Pdx1cKO mutants at P1 and P28. Expressions of Glut2 and MafA were reduced in the islets of mutant mice at P1, but reached normal level by P28 (**E**). The islet structure of Pdx1cKO mice was disrupted at P1 and P28 based on the intermingling of glucagon-expressing cells within the islet (**F**). Scale bars, 500 μm in Low Mag. pictures in (**A**), 50 μm in High Mag. pictures in (**A**) and in (**E,F**) Bars represent the mean value ± SE. *P < 0.05, ***P < 0.001.

At P1, Pdx1cKO mice formed smaller islets and the total endocrine area was 17% that of control (Fig. 6A,B). Between P1-P28, approximately 5.45-fold expansion in pancreatic endocrine cell content occurred in control mice, but only 3.57-fold in Pdx1cKO mice (Fig. 6A,B). Consequently, the endocrine content of mutant pancreata corresponded to approximately 11% that found in control mice at P28. It should be noted that the reduction in endocrine cell content was even more severe than predicted from the reduced body weight (approximately 81% that of control mice) and pancreas weight (approximately 31% that of control mice) at P28 (Fig. 1C,D). Immunolabeling for PHH3 revealed reduced proliferation of endocrine cells in the mutant pancreata at P1 (Fig. 6C), while TUNEL assays showed no significant difference (Fig. 6D).

Finally, in Pdx1cKO mice, along with the reduced pancreatic endocrine cell number, the maturation of β cells was delayed at P1, according to the reduced expression of GLUT2 and MafA. However, the production of these proteins was restored to control levels by P28 (Fig. 6E). Islet architecture was also impaired, and glucagon-producing cells and insulin-producing cells were intermingled even within the few relatively large islets that were present at P28 (Fig. 6F).

## Discussion

The pancreas is an unusual organ in that two functionally independent tissues co-exist. Accordingly, investigators have sought potential interactions between exocrine and endocrine tissues. Here, we assessed the effects exocrine tissue has on endocrine development. Previous reports have shown that adult exocrine-driven factor(s) can regulate endocrine tissue. For example, Reg1 is expressed in the exocrine pancreas but stimulates β cell proliferation in a rat regeneration model^22^. More recently, Xiao *et al.* showed that exocrine tissue extracts from normal adult mice or from a pancreatic-duct-ligated model (PDL model) induced Ngn3 expression in cultured β cells^23^. In addition, loss of exocrine-derived Sostdc, a dual BMP and Wnt inhibitor, enhances insulin secretion under metabolic stress in adult pancreas^24^. Considering that embryonic mechanisms are sometimes reactivated in the regeneration process of an injured organ, we speculated that similar exocrine-to-endocrine effects play a role in normal pancreatogenesis.

To test this theory, we needed a mouse model whose exocrine tissue is severely hypoplastic or lacking during development. We created a new mutant mouse in which *Pdx1*, an indispensable gene for pancreas formation, is depleted in an exocrine-specific manner. As expected, *Elastase-Cre*-mediated *Pdx1* inactivation caused severe exocrine defects after complete depletion of Pdx1 protein by E14.5, which is consistent with a report by Hale *et al.*^25^. However, they did not deplete *Pdx1* in an exocrine-specific manner and instead used a tetracycline-responsive transactivator to temporally control the inactivation of *Pdx1.* Furthermore, they reported that *Pdx1* depletion at E13.5 resulted in impaired pancreas formation characterized by immature acinar cell differentiation and dilated duct-like structures that resembled the exocrine defects in our Pdx1cKO mice. We believe the experimental results described in the present study are the first demonstration that selectively abrogating exocrine formation during embryonic stages reduces the proliferation and delays the maturation of endocrine cells along with altering the islet structure to cause insufficient insulin release in mice.

It has been reported that the lineage specification of pancreatic exocrine/endocrine precursor cells is regionally controlled within the developing branches of the epithelial tree. Zhou *et al.* used *Cpa1-CreER*-mediated time-specific lineage tracing to show that Pdx1^+^Ptf1a^+^cMyc^high^Cpa1^+^ multipotent progenitor cells are located at the tips of the epithelial branches at early-to-mid embryogenesis (approx. E13–14) while endocrine/duct bipotent precursors are located within the trunk epithelium^19^. Consistent with this, we previously showed that a reduced dose of *Ptf1a* caused extreme impairment of branch formation throughout embryogenesis and glucose intolerance after birth^6^. Our study also suggested that the formation of a poorly branched ductal tree during early pancreatic development caused a reduction in the number of multipotent tip cells by E14.5, resulting in a smaller endocrine precursor pool in *Ptf1a* hypomorphic mice^6^. While our *Elastase-Cre*-mediated *Pdx1* mutant mice exhibited reduced branching of the ductal tree and a smaller percentage of Ptf1a-expressing cells, the reduced branching was less severe than in *Ptf1a* hypomorphic mice. *Pdx1* depletion occurred relatively late, at around E13.5-14.5, which coincides with when tip cells lost their multipotency. We found that inactivation of *Pdx1* by the *Elastase-Cre* transgene, which occurred in the tip region, caused abnormal cell differentiation, less proliferation and elevated cell death in the trunk domain, resulting in less formation of Ngn3(+) endocrine precursors. Thus, we propose that the reduced endocrine cell number in Pdx1cKO mice is not endocrine-autonomous, but due to an unidentified factor(s) provided by the exocrine tissue that stimulates endocrine differentiation and expansion during embryonic and postnatal development. Alternatively, the reduced branching at mid-to-late developmental stages could have resulted in fewer niche locations for endocrine progenitors to form in *Elastase-Cre*-mediated *Pdx1* mutant mice.

We also provide evidence that β cell maturation is delayed in the mutant mice, as demonstrated by the decreased immunoreactivity of Glut2 and MafA in perinatal islets. Although both proteins were restored to normal levels by P28, we observed a long-lived altered islet structure. Islet dysmorphogenesis is thought to relate to islet dysfunction, as peripheral cell types intermingling with β cells might disrupt gap junctions or other intercellular coupling involved in efficient insulin release^26^. Cell death within the islets can also impair islet architecture^27^. Thus, the formation and elimination of *Pdx1*-inactivated endocrine cells during specific developmental stages could have contributed to islet dysmorphogenesis. These results warrant future studies on the mechanisms underlying the formation and maintenance of normal islet structure, especially in terms of exocrine effects on endocrine development.

## Methods

### Mice

We obtained *Elastase* promoter-driven *Cre* transgenic mice^28^, mice carrying a floxed *Pdx1* allele (*Pdx1*^*loxP*^)^27^, *Pdx1* wild-type mice (*Pdx1*^+^) and mice with *null* alleles (*Pdx1*^–^)^14^. Mating the *Elastase* promoter-driven *Cre* transgenic mice with *ROSA26r* mice^29^ enabled us to lineage-label *Elastase-Cre* transgene-expressing cells and their progeny as X-gal-positive or β-gal-positive. To evaluate exocrine defects in Pdx1cKO (*Pdx1*^*loxP/−*^;*Elastase-Cre*;*ROSA26r or Pdx1*^*loxP/loxP*^;*Elastase-Cre*;*ROSA26r*) mice (Figs 1 and 2), *Pdx1*^*loxP/*+^;*Elastase-Cre*;*ROSA26r* mice were used as controls, since *Pdx1* heterozygous mice develop almost identically to wild-type mice during embryogenesis^14^. When analyzing endocrine development and function (Figs 3, 4, 5, 6), however, *Pdx1*^+/+^;*Elastase-Cre*;*ROSA26r* mice were used as controls, since a dosage requirement for Pdx1 has been suggested in endocrine function^16^,^17^. All animal experiments were performed in accordance with the Kyoto University guidelines for animal experiments and approved by the animal research committee of Kyoto University.

### Genotyping

Genomic DNA from either mouse embryonic heads or postnatal tail tips was genotyped by PCR using the primer sets listed in Table S1.

### Tissue Preparation for X-gal Staining and Paraffin Sections

Tissue preparation, X-gal staining and paraffin sections were performed as previously described^5^.

### Immunohistochemistry and immunofluorescence

After rehydration, slides were washed and incubated for 30 min at room temperature with Protein Block (Dako) followed by overnight incubation at 4 °C with primary antibodies (Table S2). The sections were washed in phosphate-buffered saline (PBS) and incubated for 60 min with secondary antibodies (Table S3). Images were taken with a BX51 microscope (Olympus) or BZ-9000E HS All-in-one Fluorescence Microscope (Keyence).

### PHH3 staining and TUNEL assays

Mitotic activity was analyzed by immunolabeling with rabbit anti-phospho-Histone H3 (Ser10) (Millipore). TUNEL assays were done using the DeadEnd Fluorometric system (Promega) according to the manufacturer’s instructions.

### Cell Counting

Cell numbers were counted at ×200 magnification in three randomly selected sections per pancreas, and the proportion of the desired cell types to all epithelial cells was calculated. To evaluate the relative endocrine area, the whole pancreas was sectioned in 3 μm intervals, and the number of Chromogranin-A(+) cells was counted in every hundredth slide.

### Intraperitoneal glucose tolerance test (IPGTT)

After overnight fasting, we measured blood glucose before- and 15, 30, 60 and 120 minutes after D-glucose injection (2 mg/g body weight, intraperitoneally) by a Glucocard DIA meter (GT1641) (Arkray). To evaluate insulin release 15 min after glucose challenge, blood was centrifuged in the presence of aprotinin (100 kIE/ml blood; Wako Chemicals), and serum insulin was measured with a mouse insulin ELISA kit (U-type) (Shibayagi).

### Statistical Analysis

All indices were analyzed using the t-test (two-tailed).

## Additional Information

**How to cite this article**: Kodama, S. *et al.* Diabetes Caused by *Elastase-Cre*-Mediated *Pdx1* Inactivation in Mice. *Sci. Rep.* **6**, 21211; doi: 10.1038/srep21211 (2016).

## Supplementary Material

Supplementary Information
