# Supplementary material for: Diabetes Caused by Elastase-Cre-Mediated Pdx1 Inactivation in Mice
Source: Sci Rep. 2016 Feb 18;6:21211. doi: 10.1038/srep21211 (PMC4758062; doi:10.1038/srep21211)
Supplement: Supplementary Information [file srep21211-s1.pdf]

Supplementary Information

Diabetes Caused by *Elastase-Cre*-Mediated *Pdx1* Inactivation in Mice

Sota Kodama, Yasuhiro Nakano, Koji Hirata,
Kenichiro Furuyama, Masashi Horiguchi, Takeshi Kuhara,
Toshihiko Masui, Michiya Kawaguchi, Maureen Gannon,
Christopher V.E. Wright, Shinji Uemoto
and Yoshiya Kawaguchi

Pdx1$^{+/+}$;Elastase-Cre;R26r

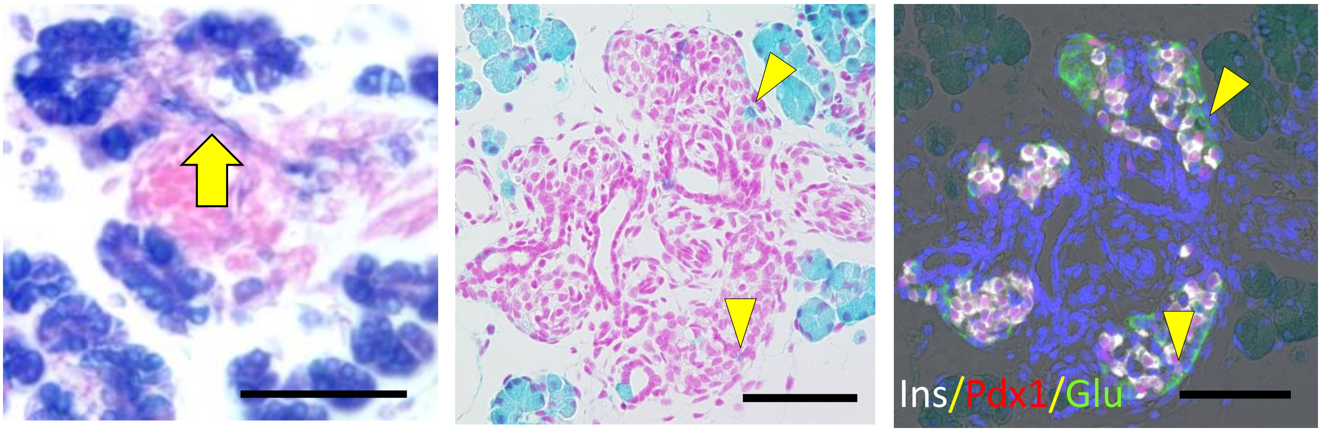

Figure S1

Assessment of the specificity and efficiency of Elastase-Cre mediated recombination

Cell tracking of the *Elastase-Cre*-expressing cells. Up to 100% of acinar cells, a subset of small ducts connected to acini (arrow) and very few endocrine cells (arrowheads) are labeled in *Pdx1*$^{+/+}$;*Elastase-Cre*;*R26r* mice at P1. Middle and Right panels are serial sections. Scale bars, 100 μm.

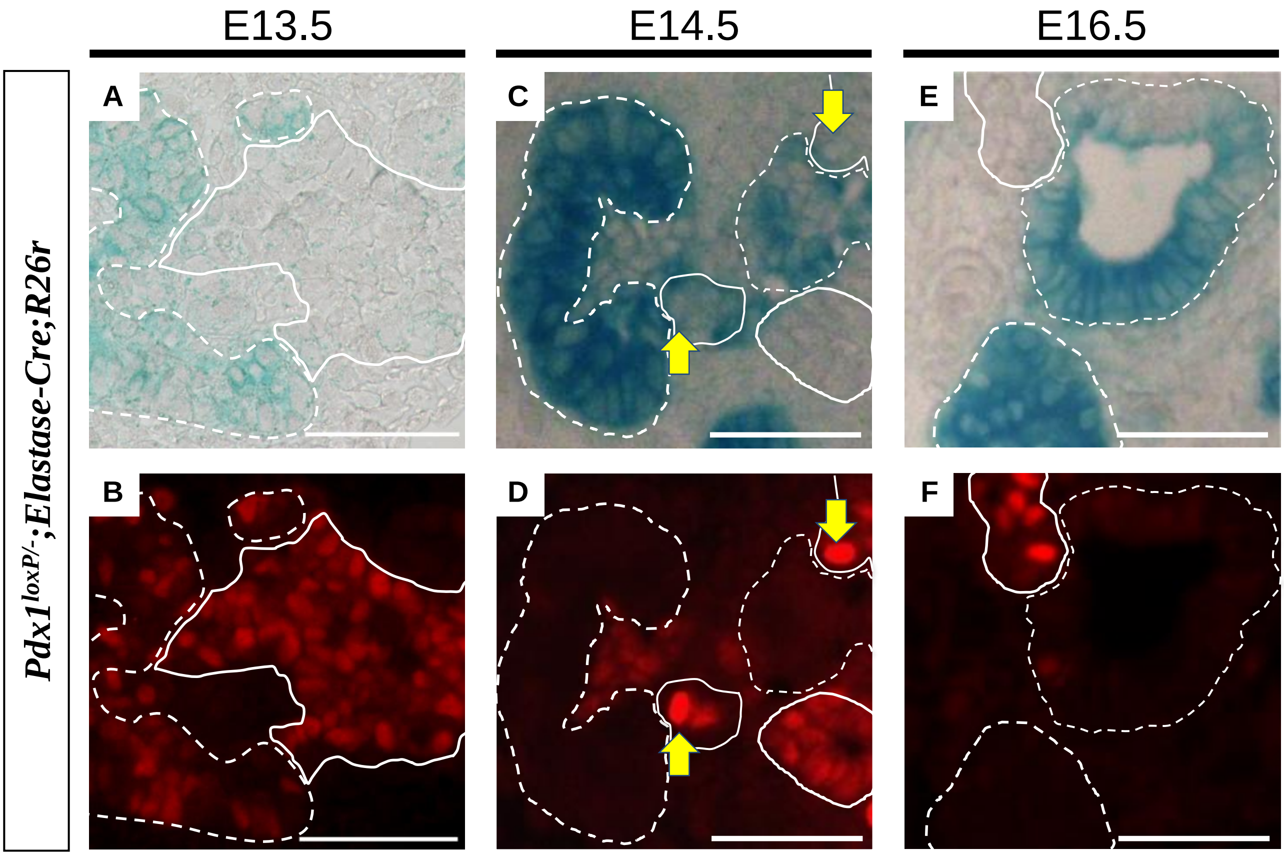

Figure S2

Elastase-Cre-mediated Pdx1 inactivation and lineage tracing

In the *Pdx1*$^{loxP/-}$;*Elastase-Cre*;*R26r* mice, X-gal-positive cells (blue, surrounded by dotted lines) do not costain for Pdx1(red) at E14.5 and E16.5, confirming the successful recombination of the ROSA26r allele and the floxed *Pdx1* allele, but X-gal-negative cells (surrounded by solid lines or indicated by arrows) retain Pdx1 expression. Note that some X-gal-positive cells retain faint Pdx1 expression at E13.5. A and B, C and D, E and F are the same sections, respectively. Scale bars, 50 μm (*A*–*D*).

Table S1. Genotyping PCR primers sets

		Sequence
Pdx1 wild-type allele	Forward	5′–CCTTTGCGGATCCTT–3′
	Reverse	5′–GCCAACAACTGGCAGATTC–3′
Pdx1 $^{-}$ allele	Forward	5′–CAAGCCAGGGAGTTAAATC–3′
	Reverse	5′–AGGTGAGATGACAGGAGATC–3′
Pdx1 ^{loxP} allele	Forward	5′–AGGGTTCCGGATCGATCCCC–3′
	Reverse	5′–AGCAGCTGGAGCAGGTAGGC–3′
Elastase-Cre	Forward	5′–ACCTGAAGATGTTCGCGATTATCT–3′
	Reverse	5′–ACCGTCAGTACGTGAGATATCTT–3′
ROSA26r	Forward	5′–ATCCTCTGCATGGTCAGGTC–3′
	Reverse	5′–CGTGGCCTGATTCATTCC–3′

Table S2. Primary Antibodies

Antigen	Species	Dilution	Supplier (Cat. #)
Pdx1	Goat	1:15000	C.V.E. Wright, Vanderbilt University
Amylase	Rabbit	1:400	Sigma-Aldrich, Missouri, USA (A8273)
Cytokeratin	Mouse	1:100	Dako, Glostrup, Denmark (M3515)
Insulin	Guinea pig	1:300	Dako, Glostrup, Denmark (A0564)
Glucagon	Rabbit	1:300	Dako, Glostrup, Denmark (A0565)
PHH3	Rabbit	1:300	Upstate, New York, USA (04-1093)
MafA	Rabbit	1:100	Bethyl, Alabama, USA (IHC-00352)
Chromogranin A	Goat	1:400	Santa Cruz, California, USA (sc-1488)
Glut2	Goat	1:100	Santa Cruz, California, USA (sc-7580)
Nkx6.1	Goat	1:100	R&D systems, Minnesota, USA (AF5857)
β-Galactosidase	Rabbit	1:4000*	Cappel, California, USA (55976)
Ngn3	Mouse	1:300	DSHB, Iowa, USA (F25A1B3)
Ptf1a	Rabbit	1:15000*	Mikio Hoshino, National Institute of Neuroscience
Hnf1β	Mouse	1:500*	BD Biosciences, California, USA (612504)

*: TSA system (PerkinElmer) was used in accordance with the manufacturer's instructions.

Table S3. Secondary Antibodies

Conjugate	Antigen	Species	Dilution	Supplier
Cy3	Goat IgG	Donkey	1:400	Chemicon, Massachusetts, USA
Cy3	Rabbit IgG	Donkey	1:400	Chemicon, Massachusetts, USA
Cy3	Guinea pig IgG	Goat	1:400	Chemicon, Massachusetts, USA
Alexa Fluor 488	Mouse IgG	Goat	1:1000	Molecular Probes, California, USA
Alexa Fluor 488	Guinea pig IgG	Goat	1:1000	Molecular Probes, California, USA
Alexa Fluor 488	Goat IgG	Donkey	1:1000	Molecular Probes, California, USA
Alexa Fluor	Guinea pig IgG	Goat	1:200	Molecular Probes, California, USA
HRP (HISTOFINE)	Goat IgG	Rabbit	1	Nichirei Bioscience, Tokyo, Japan
HRP (ImmuPress)	Mouse IgG	Horse	1	Vector Laboratories, California, USA
HRP (ImmuPress)	Rabbit IgG	Horse	1	Vector Laboratories, California, USA
